# 1,25(OH)_2_D_3_ downregulates the Toll-like receptor 4-mediated inflammatory pathway and ameliorates liver injury in diabetic rats

**DOI:** 10.1007/s40618-015-0287-6

**Published:** 2015-04-24

**Authors:** H. Wang, Q. Zhang, Y. Chai, Y. Liu, F. Li, B. Wang, C. Zhu, J. Cui, H. Qu, M. Zhu

**Affiliations:** Department of Endocrinology, Tianjin Medical University General Hospital, Tianjin, 300052 China; Division of Epidemiology, Human Genetics and Environmental Sciences, University of Texas School of Public Health, Houston, TX 77030 USA

**Keywords:** Diabetes, 1,25(OH)_2_D_3_, Inflammation, Toll-like receptor 4, Hepatocytes, Liver injury

## Abstract

**Background:**

Fatty acid deposition in the liver can activate a number of pro-inflammatory signaling pathways such as the Toll-like receptor 4 (TLR4) pathway, which may be important in the pathogenesis of nonalcoholic steatohepatitis. 1,25(OH)_2_D_3_ downregulates the expression of TLR4 and may represent a novel treatment strategy for reducing hepatocyte injury. Therefore, in this study, we investigated the protective effects of 1,25(OH)_2_D_3_ on diabetic liver injury in vivo.

**Methods:**

Streptozotocin (STZ)-induced diabetic rats were randomly divided into five groups and treated with low-dose 1,25(OH)_2_D_3_ (0.025 μg/kg/day), medium-dose 1,25(OH)_2_D_3_ (0.15 μg/kg/day), high-dose 1,25(OH)_2_D_3_ (0.3 μg/kg/day), insulin (protamine zinc insulin 16 U/kg/day, subcutaneous injection), or no intervention (the control group). Sixteen weeks later, the rats were killed, and blood samples were obtained to test lipid profiles and hepatic function. The infiltration of inflammatory cells, the level of fibrosis, and the expression levels of TLR4, nuclear factor-kappa B (NF-κB), and tumor necrosis factor-α (TNF-α) in the liver were analyzed. The hepatocytes were treated with vehicle control, LPS (100 ng), high fat [DMEM + FFA (0.1 mM: palmitic acid, oleic acid, 1:2)], LPS + high fat, vehicle + 1,25(OH)_2_D_3_ (10^−7^ M), LPS + 1,25(OH)_2_D_3_, high fat + 1,25(OH)_2_D_3_, or LPS + high fat + 1,25(OH)_2_D_3_. RNA and protein were extracted to detect the expression of TLR4 and downstream inflammatory factors such as NF-ΚB, TNF-α, and IL-6. Groups of data were compared by single factor variance analysis.

**Results:**

High-dose 1,25(OH)_2_D_3_ administration for 16 weeks downregulated the expression of TLR4, NF-κB, and TNF-α in the liver tissue of diabetic rats and attenuated hepatic inflammation and fibrosis, as shown by immunohistochemical staining, hematoxylin and eosin staining, Masson’s trichrome staining, reverse transcription polymerase chain reaction (RT-PCR), and western blotting. In vitro, hepatocytes treated with high fat or LPS exhibited significantly increased expression of TLR4, NF-κB, and downstream inflammatory factors (*P* < 0.05). Intervention with 1,25(OH)_2_D_3_ decreased the expression of TLR4, NF-κB, and inflammatory factors (*P* < 0.05).

**Conclusions:**

1,25(OH)_2_D_3_ exhibited protective effects against diabetes-related liver injury, possibly through downregulation of components of the TLR4 signaling pathway.

## Introduction

Diabetes mellitus (DM) is a serious disease in which chronic complications can cause multiple organ injuries, including damage to the liver [1]. Accumulation of fatty acids, particularly saturated fatty acids in the liver can activate a number of pro-inflammatory signaling pathways, including the Toll-like receptor (TLR) pathway [2]. Unlike other TLRs, TLR4 can bind to endogenous ligands associated with cell injury, such as inflammatory mediators/factors [2, 3]. Additionally, the TLR4 signaling pathway can activate nuclear factor-kappa B (NF-κB) and induce the expression of pro-inflammatory genes. These changes promote recruitment and activation of inflammatory cells, thereby resulting in the activation of inflammation and fibrosis [4, 5].

Studies have shown that TLR activation and vitamin D deficiency play important roles in the pathogenesis of type 1 diabetes mellitus (T1DM) [6, 7]. Indeed, the expression of TLR4 is increased in monocytes from patients with T1DM compared with healthy controls [6]. Moreover, patients with T1DM and T1DM with microvascular complications are significantly vitamin D deficient compared with control subjects [7]. Interestingly, there is a negative correlation between TLR4 expression and serum concentration of 25(OH)D_3_. In monocytes from patients with T1DM pretreated for 24 h with 1,25(OH)_2_D_3_ (0.1 µM), the expression of TLR4 and cytokines in response to lipopolysaccharide is decreased [7]. However, few studies have investigated the effects of vitamin D on nonalcoholic fatty liver disease (NAFLD) in vivo, either by sunlight therapy or by vitamin D supplementation to the diet in order to increase the serum level of 25(OH)D_3_. To avoid the effects of unstable vitamin D on serum vitamin D levels and to prevent the hydroxylation of vitamin D to 1,25(OH)_2_D_3_ both in the liver and kidney, we used 1,25(OH)_2_D_3_, the active form of vitamin D in vivo, and investigated the protective effects of 1,25(OH)_2_D_3_ on diabetic liver injury.

## Materials and methods

### Chemicals

Streptozotocin (STZ) and 1,25(OH)_2_D_3_ were purchased from Sigma–Aldrich (St. Louis, MO, USA). Polyclonal antibodies targeting rat TLR4, TNF-α, NF-κB p65, phospho-NF-κB, and β-actin were purchased from Cell Signaling Technology (Danvers, MA, USA). Protamine zinc insulin (P) was obtained from Jiangsu Wanbang Biological Technology Ltd. (Xuzhou, Jiangsu, China). CD68 was purchased from Wuhan Boster Biological Technology Ltd. (Wuhan, Hubei, China).

### Animals and treatments

All experimental procedures were performed in accordance with the National Institutes of Health Guide for the Care and Use of Laboratory Animals and were approved by the Animal Care and Use Committee of Tianjin Medical University (Tianjin, China). Ninety 8-week-old male Sprague–Dawley (SD) rats, with body weights ranging from 180 to 220 g, were obtained from Beijing Huafukang Biological Science and Technology Stock Co. Ltd. (Beijing, China). The animals were maintained in a room with controlled temperature (21 ± 2 °C) and a 12:12-h light–dark cycle, with free access to food pellets (standard rat chow) and tap water. Eighty rats were given single injections of STZ (45 mg/kg) into the tail vein. Seventy-five of these rats subsequently acquired diabetic symptoms, having blood glucose concentrations greater than 16.7 mM, and underwent further treatment for 16 weeks. The remaining ten rats that did not receive STZ injection were treated with 0.1 M sodium citrate buffer as vehicle and were designated as the normal control group (Group C).

Diabetic rats were divided randomly into five groups: Group D, with untreated diabetes mellitus; Group H, treated with high-dose 1,25(OH)_2_D_3_ (0.3 µg/kg/day); Group M, treated with medium-dose 1,25(OH)_2_D_3_ (0.15 µg/kg/day); Group L, treated with low-dose 1,25(OH)_2_D_3_ (0.025 µg/kg/day); and Group I, treated with insulin (protamine zinc insulin, 16 U/kg/day, subcutaneous injection). 1,25(OH)_2_D_3_ was administrated daily by oral gavage in rats at 09:00 h. Standard rat chow, containing 48 % corn, 20 % soybean meal, 10 % wheat bran, 10 % flour, 8 % fish meal, 2 % farina, 1 % vegetable oil, 0.5 % salt, and 0.5 % trace elements, was provided to all groups of rats.

### Biochemical and histological analysis

Sixteen weeks after induction of diabetes, all rats were weighed and euthanized. The day before euthanasia, the rats were fasted overnight from 20:00 h, and blood samples were obtained at 08:00 h on the following day. Analysis of glucose, alanine aminotransferase (ALT), C-reactive protein (CRP), total cholesterol (TC), triglycerides (TG), low-density lipoprotein (LDL), calcium, and phosphorus in plasma samples was performed using standard enzymatic techniques (Hitachi 7600 Automatic Biochemistry Analyzer, Hitachi, Japan). After euthanasia, the livers were removed, and half of each liver was fixed in 10 % buffered formalin and embedded in paraffin. Formalin-fixed liver tissue was processed for histological analysis, including immunohistochemical staining with different antibodies or hematoxylin and eosin staining (H&E) or Masson’s trichrome staining to detect collagen for evaluation of fibrosis. The remaining liver tissues were stored at −80 °C until use for RNA and protein extraction and subsequent reverse transcription polymerase chain reaction (RT-PCR) and western blotting analyses.

### Isolation of hepatocytes

Four-month-old male SD rats (150–200 g) were purchased from Beijing Huafukang Biological Science and Technology Stock Co. Ltd. (Beijing, China). A vertical incision on the left side of abdomen was made to expose the portal vein and inferior vena cava. Then, a 24-gauge catheter was inserted into the portal vein for perfusion. Hepatocytes were isolated from the livers of SD rats with HBSS (5.33 mM KCl, 0.44 mM KH_2_PO_4_, 0.34 mM Na_2_HPO_4_, 138 mM NaCl, and 4.17 mM NaHCO_3_, adjusted to pH 7.5–8.0) containing 0.5 mM EDTA to remove blood. Cells were then treated with 0.05 % collagenase IV in Dulbecco’s modified Eagle’s medium (DMEM, 5.5 mM glucose; Invitrogen, USA). Primary hepatocytes were cultured at a density of 2–6 × 10^5^ cells per dish in DMEM supplemented with 5 % fetal bovine serum (Gibco, USA), 1 % l-glutamine, 1 % nonessential amino acids, and 1 % penicillin–streptomycin (Invitrogen, USA) at 37 °C in an atmosphere containing 5 % CO_2_. After incubation for 24 h, cells were adherent and split in six-well plates. Primary hepatocytes were divided randomly into seven groups: Group C, normal control group; Group L, treated with LPS (Sigma, USA) (100 ng); Group F, treated with DMEM containing high fat and FFAs (0.1 mmol, palmitic acid: oleic acid = 1:2; Sigma, USA); Group L + F, treated with LPS and high fat; Group L + VD, treated with LPS and 1,25(OH)_2_D_3_ (10^−7^ M; Sigma); Group F + VD, treated with high fat and 1,25(OH)_2_D_3_; and Group L + F + VD, treated with LPS, high fat, and 1,25(OH)_2_D_3_. Cells were harvested for quantitative RT-PCR and western blot analysis.

### RT-PCR

Total RNA was prepared using TRIzol reagent (Invitrogen, Carlsbad, CA, USA), according to the manufacturer’s instructions. RNA was reverse-transcribed into complementary DNA (cDNA) by using a SuperScript First-Strand Synthesis System for RT-PCR Kit (Invitrogen), and the cDNA fragments were amplified by PCR with Taq DNA polymerase (Invitrogen). PCR was carried out in a thermal cycler (Bio-Rad, Hercules, CA, USA). Initial denaturation was carried out at 95 °C for 15 min, followed by 40 cycles of denaturation for 10 s at 95 °C, annealing for 30 s at the appropriate temperature, and extension for 32 s at 72 °C. The primer sequences used for PCR were as follows: *β*-*actin*, forward 5′-CCTCATGCCATCCTGCGTCTG-3′ and reverse 5′-TTGCTCGAAGTCTAGGGCAACATAG-3′ (annealing temperature: 58 °C); *TLR4*, forward 5′-GCCTTGAATCCAGATGAAAC-3′ and reverse 5′-CTGTGAGGTCGTTGAGGTTAG-3′ (annealing temperature: 58 °C); *NF*-*κB*, forward 5′-TCCCCTGTACGATAGTCGGCTC-3′ and reverse 5′-GAGCGTTGCTTTGGATCAAGG-3′ (annealing temperature: 60 °C); *TNF*-*α*, forward 5′-GAAAAGCAAGCAACCAGCCA-3′ and reverse 5′-CGGATCATGCTTTCCGTGCTC-3′ (annealing temperature: 57 °C); and *IL*-*6*, forward 5′-CTGGCAATATGAATGTTGAAAC-3′ and reverse 5′-AGAAACCATCTGGCTAGGTAAG-3′ (annealing temperature: 58 °C).

### Western blotting

The levels of TLR4, TNF-α, NF-κB p65, and phospho-NF-κB in liver tissue and hepatocytes were analyzed by western blotting. Proteins were extracted using RIPA buffer (Thermo, USA), according to the manufacturer’s instructions, and the protein concentrations were measured using a BCA protein assay kit (Thermo, USA). An equal amount of protein from each sample was subjected to sodium dodecyl sulfate (SDS)-polyacrylamide gel electrophoresis and then transferred to polyvinylidene difluoride (PVDF) membranes (Bio-Rad). Membranes were blocked with 5 % milk in 1 × Tris-buffered saline with 0.1 % Tween-20 (TBST) for 1 h at room temperature and incubated overnight at 4 °C with primary antibodies. Membranes were washed twice for 10 min in 1 × TBST and then incubated with horseradish peroxidase (HRP)-conjugated secondary antibodies for 2 h. Membranes were then washed twice for 10 min in 1 × TBST. Proteins were visualized by ECL (Millipore), and blots were scanned using a Fluor-S MAX MultiImager System (Bio-Rad). The signal intensities were determined using Quantity One image software (Bio-Rad).

### Statistical analysis

Normally distributed data were expressed as the mean ± standard deviation (SD). Statistical analysis was performed using analysis of variance (ANOVA) for multiple group comparisons. SPSS 19.0 statistical software was used for data analysis, and differences with *P* values less than 0.05 were considered significant.

## Results

### Plasma concentrations of biochemical parameters in rats

Biochemical parameters for the different groups of rats are shown in Table 1. Compared with Group C, the body weights and liver weights of diabetic rats in Groups D, L, M, and H were significantly decreased. Additionally, glucose levels were higher in rats in Group D than in those of the other groups, demonstrating that rats administered 1,25(OH)_2_D_3_ or insulin exhibited reduced blood glucose levels. ALT levels in rats of all five diabetic groups were higher than those in rats of Group C. Moreover, compared with rats in Group D, rats in Groups H and I exhibited significantly reduced ALT levels (*P* < 0.05). Compared with the control group, levels of ALB in diabetic rats decreased significantly (*P* < 0.05). Interestingly, serum calcium tended to increase in the three groups of rats treated with 1,25(OH)_2_D_3_; however, these differences were not significant (*P* > 0.05). The same trend was observed for serum phosphate. Compared with Group C rats, Group D rats showed an increase in serum CRP levels (*P* < 0.05). Moreover, compared with rats in Group D, rats in Groups H and M showed significantly reduced CRP levels (*P* < 0.05). No significant differences were observed for TC levels in all groups of rats (*P* > 0.05); however, TC levels tended to decrease in rats treated with 1,25(OH)_2_D_3_. In addition, compared with the levels of TG in Group C rats, those in Group D rats were significantly increased (*P* < 0.05), while those in Group H rats were significantly decreased (*P* < 0.05). Finally, rats in Group D showed significantly higher levels of LDL than did rats in Group C, and LDL levels were dramatically decreased in rats treated with 1,25(OH)_2_D_3_, particularly in rats of Groups M and H (*P* < 0.05).

**Table 1:** Biochemical parameters for rats evaluated in this study

	Groups					
	C (*n* = 10)	C (*n* = 10)	C (*n* = 10)	C (*n* = 10)	C (*n* = 10)	C (*n* = 10)
Body weight (g)	440.70 ± 9.89^#,▼^	289.50 ± 23.51*^,▼^	289.10 ± 19.66*^,▼^	293.50 ± 22.04*^,▼^	302.30 ± 18.83*^,▼^	399.90 ± 27.16*^,#^
Liver weight (g)	12.49 ± 2.47^#,▼^	6.66 ± 0.79*	6.93 ± 0.62*	7.85 ± 0.85*	8.21 ± 1.61*	8.1 ± 0.65*
Glucose (mM)	6.24 ± 0.50 ^#^	29.00 ± 4.22*^,▼^	26.93 ± 3.80*^,▼^	24.73 ± 3.53*^,#,▼^	21.98 ± 1.19*^,#,▼^	7.75 ± 0.96 ^#^
ALB (g/L)	44.4 ± 4.69^#^	31.7 ± 5.08*^,▼^	43.0 ± 6.18^#^	41.8 ± 4.54^#^	44.8 ± 6.18^#^	41.3 ± 6.58^#^
ALT (U/L)	49.5 ± 8.07^#,▼^	83.3 ± 13.8*^,▼^	76.7 ± 8.38*^,▼^	89.8 ± 13.4*^,▼^	70.3 ± 13.1*^,#^	64.4 ± 13.8*^,#^
Serum calcium (mM)	2.28 ± 0.24	2.24 ± 0.43	2.41 ± 0.25	2.46 ± 0.23	2.32 ± 0.26	2.34 ± 0.33
Serum phosphate (mM)	2.53 ± 0.42	2.66 ± 0.25	2.43 ± 0.11	2.63 ± 0.12	2.46 ± 0.41	2.51 ± 0.11
CRP (mg/dL)	1.08 ± 0.14^#^	1.32 ± 0.17*	1.2 ± 0.23	1.06 ± 0.08^#^	1.06 ± 0.18^#^	1.18 ± 0.15
TG (mM)	0.79 ± 0.20^#,▼^	1.29 ± 0.19*	1.30 ± 0.30*	1.15 ± 0.34*	0.91 ± 0.29^#^	1.10 ± 0.30*
TC (mM)	1.06 ± 0.08	1.50 ± 0.38	1.22 ± 0.28	1.32 ± 0.49	1.06 ± 0.28	1.18 ± 0.27
LDL (mg/dL)	1.51 ± 0.26^#,▼^	2.81 ± 0.50*	2.44 ± 0.29*	2.11 ± 0.38*^,#,▼^	1.71 ± 0.27^#,▼^	2.73 ± 0.48*

*C* control, *D* diabetes, *L* low dose, *M* medium dose, *H* high dose, *I* insulin

* Compared with Group C, *P* < 0.05

^#^Compared with Group D, *P* < 0.05

^▼^Compared with Group I, *P* < 0.05

### Histological findings of liver tissues in rats

**Fig. 1:**
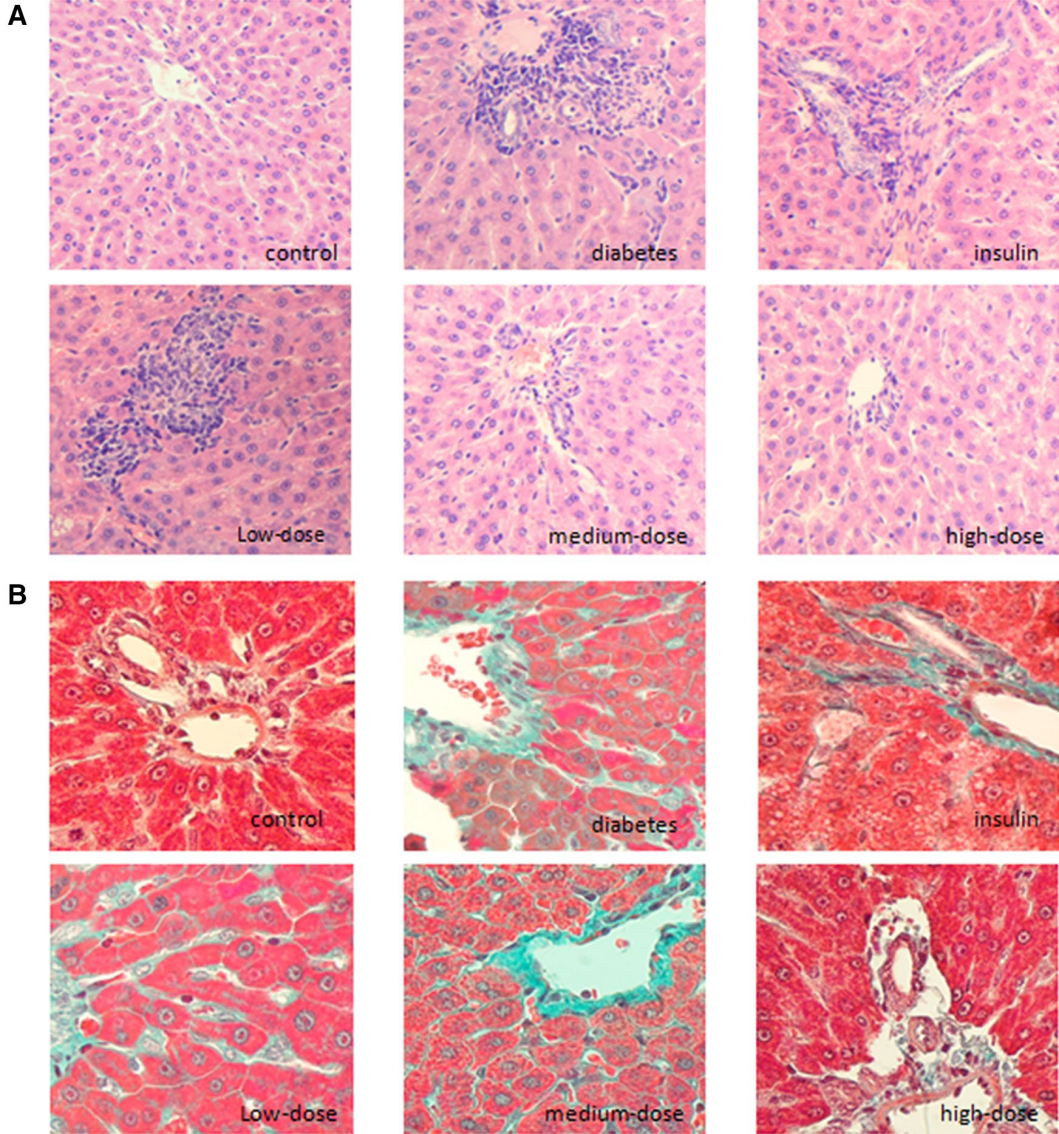
1,25(OH)_2_D_3_ exhibited protective effects in livers from diabetic rats. **a** H&E staining (×200) showed obvious inflammatory cell infiltration around hepatocytes and diffuse infiltration around the portal vein in diabetic rats. Infiltration of inflammatory cells with steatosis of hepatocytes was observed in diabetic rats treated with insulin. Alleviation of the infiltration of inflammatory cells was observed in diabetic rats treated with medium- and high-dose 1,25(OH)_2_D_3_. **b** Masson’s trichrome staining (×400) showed colorization around perisinusoidal spaces, cells, and the portal area, and the structure of the hepatic lobule was abnormal in diabetic rats. The level of interstitial fibrosis decreased, and the structure of the liver was intact in diabetic rats treated with high-dose 1,25(OH)_2_D_3_

As shown in H&E-stained sections in Fig. 1, there was no obvious inflammatory cell infiltration in Group C, and only minor punctate changes in the portal area were observed. Obvious inflammatory cell infiltration around hepatocytes, degeneration of hepatocytes, and diffuse infiltration around the portal vein were observed in Group D. In Group I, infiltration of inflammatory cells was observed, accompanied by different levels of hepatic steatosis, although not to the extent observed in Group D. Lesions in Group L were similar to those in Group D. However, interestingly, Group H exhibited reduced infiltration of inflammatory cells and a clear arrangement and structure of hepatocytes. The infiltration of inflammatory cells in Group M was less than that in Group L, but more than that in Group H, consistent with the dose of vitamin D (Fig. 1a).

Using Masson’s trichrome staining (Fig. 1b), weak signal accumulation (green coloration) appeared along the vascular wall of the portal vein and portal area in Group C. However, no obvious coloration was observed around sinusoidal spaces and cells. In contrast, staining was observed around sinusoidal spaces and cells in the portal area in Group D. Moreover, the structure of the hepatic lobule was abnormal. In Group I, the level of interstitial fibrosis decreased, and fibrosis was mainly present around sinusoidal spaces. Tissues from Group L resembled those from Group D, and the level of fibrosis observed in tissues from Group M was much lower than that in Group L. Finally, the lowest level of fibrosis was observed in Group H, in which the liver structure was normal and intact (Fig. 1b).

### Immunohistochemical staining

**Fig. 2:**
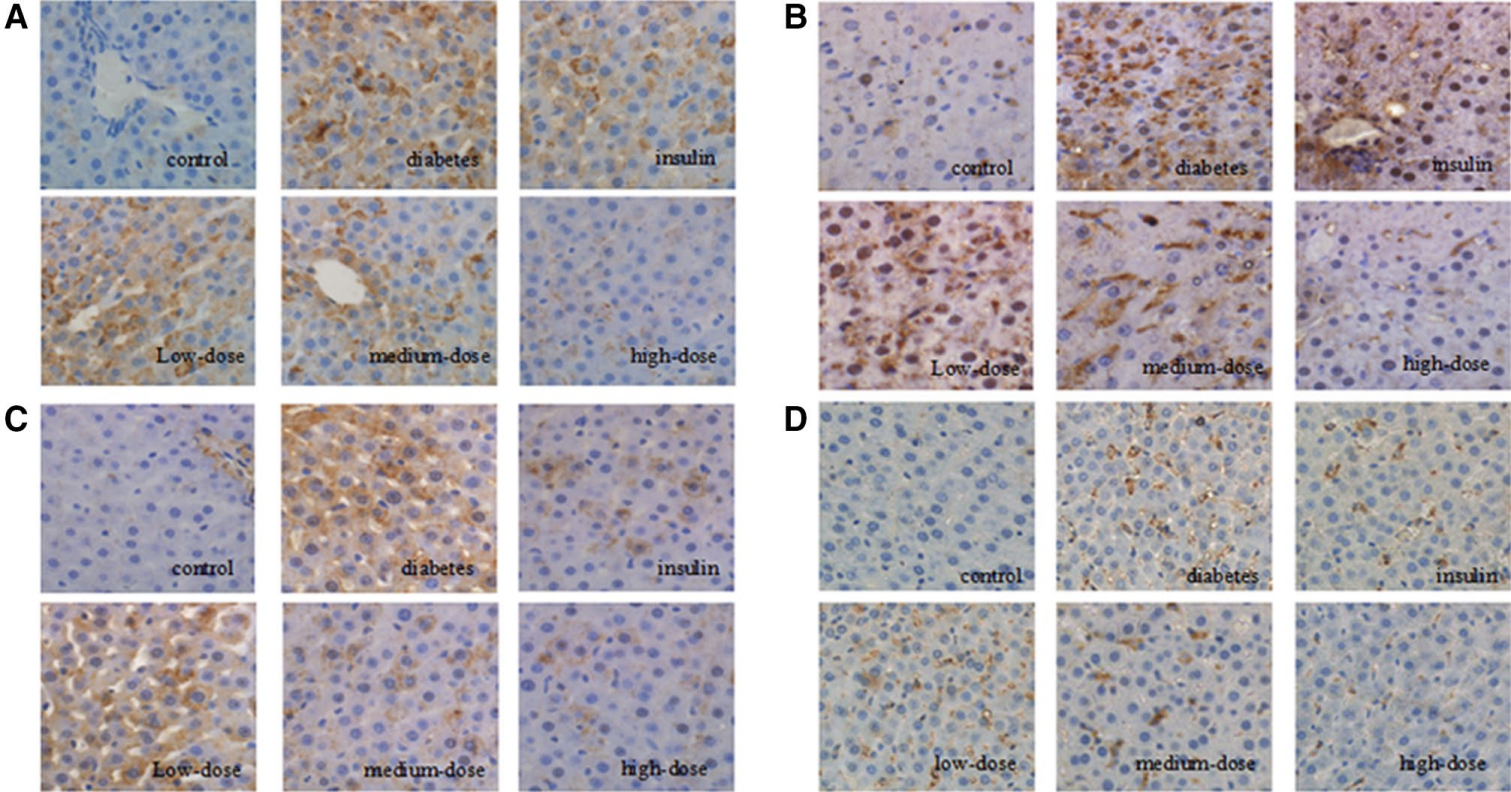
1,25(OH)_2_D_3_ administration downregulated TLR4, NF-κB, TNF-α, and CD68 proteins in the liver (magnification, ×200). **a** TLR4 staining was observed in the cytoplasm of hepatocytes and Kupffer cells in diabetic rats, with reduced staining observed in rats treated with high-dose 1,25(OH)_2_D_3_. **b** NF-κB staining was observed in the nuclei of hepatocytes and Kupffer cells in diabetic rats. However, minimal, discrete staining was observed in Kupffer cells in the hepatic sinusoids in rats treated with high-dose 1,25(OH)_2_D_3_. **c** TNF-α staining showed brown deposition on the surfaces of hepatocytes and Kupffer cells in diabetic rats. The number of positively stained cells decreased, and the staining became lighter in rats treated with high-dose 1,25(OH)_2_D_3_. **d** CD68 staining showed that there were more and larger Kupffer cells in diabetic control (untreated) rats than in rats treated with insulin or medium- or high-dose 1,25(OH)_2_D_3_

Next, we examined the immunohistochemical staining patterns of TLR4 components in tissues from each group of rats. TLR4 expression was observed only in a few hepatocytes in Group C tissues. However, TLR4 expression was observed on the membranes of hepatocytes and Kupffer cells in Group D tissues. Tissues from Group L also exhibited upregulated TLR4 expression. However, decreased expression (recovery toward the levels observed in Group C) was observed for Groups I, M, and H (Fig. 2a).

Similar patterns of expression were observed for NF-κB, with low expression observed in the nuclei of Kupffer cells in hepatic sinusoids in Group C and increased expression in hepatocyte nuclei and Kupffer cells in Group D. The expression of NF-κB in hepatocyte nuclei was also high in Groups L and I, while minimal expression was observed in some Kupffer cells in hepatic sinusoids in Group M. No clear expression of NF-κB was observed in Group H (Fig. 2b).

Lastly, patterns of TNF-α and CD68 expression were also similar to those of TLR4, with low expression in Group C, increased expression in Groups D and L, and reduced expression in Groups M, I, and H (Fig. 2c, d).

Therefore, taken together, the results of immunohistochemical analysis indicated that components of the TLR4 pathway were downregulated by 1,25(OH)_2_D_3_ treatment of diabetic rats.

### RT-PCR and western blotting analysis

**Fig. 3:**
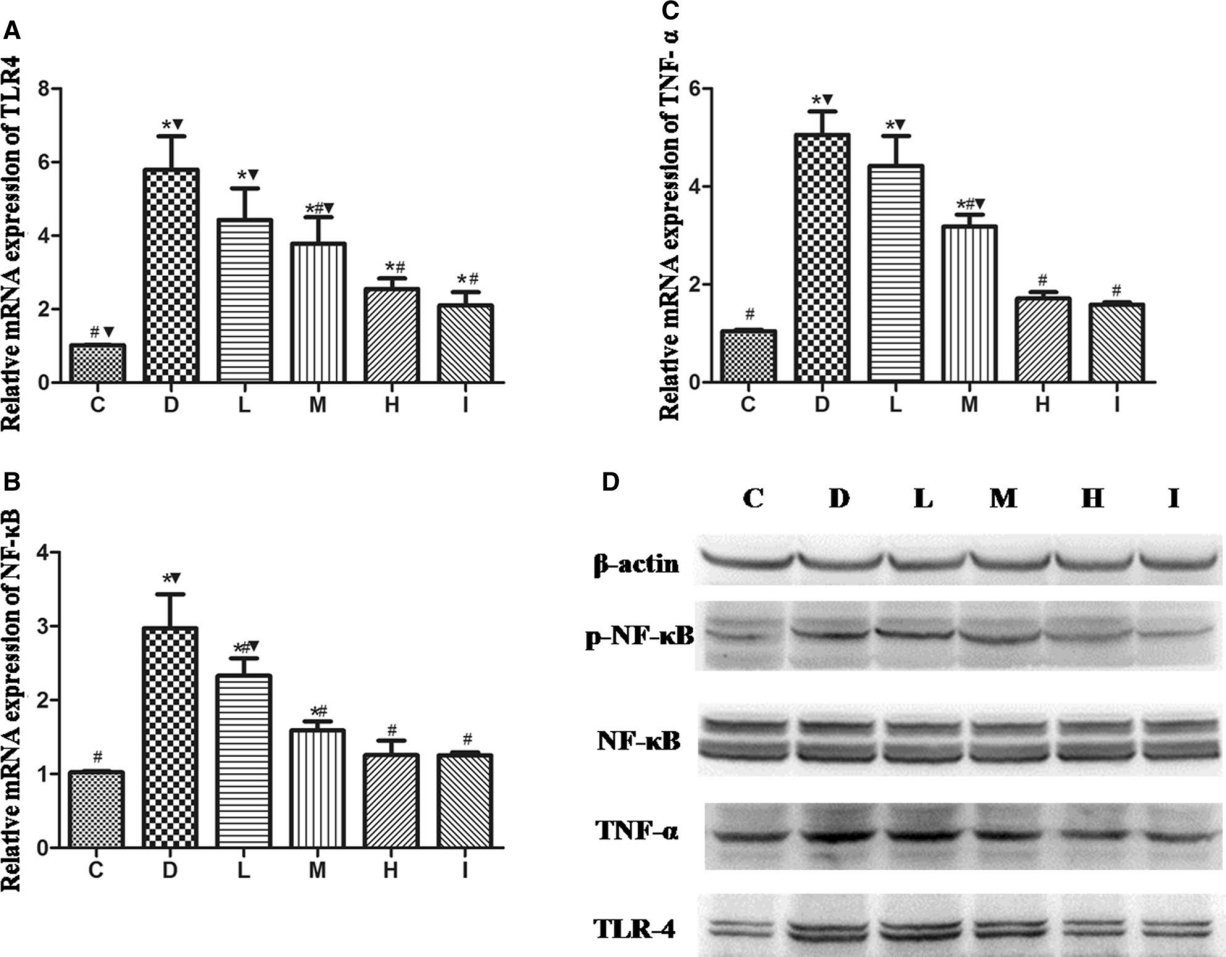
1,25(OH)_2_D_3_ downregulated the expression of components of the TLR4 signaling pathway. The relative mRNA expression levels of *TLR4*, *NF*-*κB*, and *TNF*- *α* were measured in all groups of rats, and changes in expression relative to Group C were determined (**a**–**c**). Western blotting was used to assess the levels of TLR4, phospho-NF-κB, and TNF-*α* in all groups of rats (**d**). *Compared with Group C, *P* < 0.05; ^#^compared with Group D, *P* < 0.05; ^▼^compared with Group I, *P* < 0.05

We investigated the mRNA levels of TLR4 signaling pathway components in the liver tissues. *TLR4*, *NF*-*κB*, and *TNF*-*α* mRNA levels in the liver tissues of rats in Group D were increased compared with those in Group C. Additionally, levels of all transcripts were significantly decreased (*P* < 0.05) in rats in Groups M, H, and I as compared with those in rats in Group D. Moreover, comparison of transcript levels in Groups M, H, and L demonstrated that 1,25(OH)_2_D_3_ treatment decreased gene expression in a dose-dependent manner (Fig. 3a–c). Furthermore, TLR4, phospho-NF-κB 65, and TNF-α levels were increased in Group D compared with Group C and in Groups H and I compared with Group D (Fig. 3d).

**Fig. 4:**
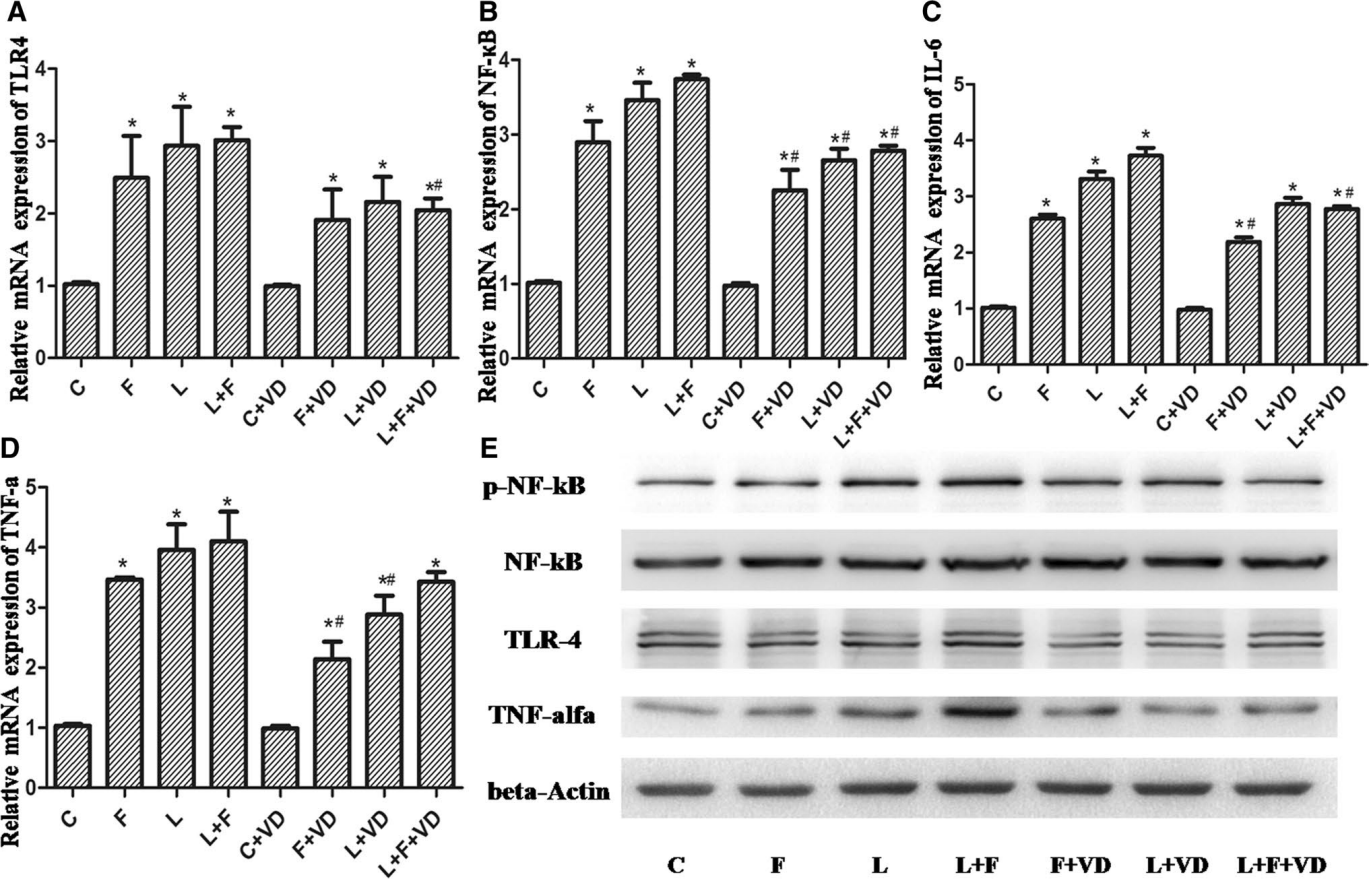
*TLR4*, *NF*-*κB*, *IL*-*6*, and *TNF*-*α* mRNA levels in hepatocytes. **a**–**d** The mRNA expression levels of *TLR4*, *NF*-*κB*, *IL*-*6*, and *TNF*-*α* were examined for the different treatment groups. **e** Protein expression of TLR4 and TNF-α and phosphorylation levels of NF-κB 65 were examined by western blotting

Primary hepatocytes were harvested for RT-PCR or western blot analysis. *TLR4*, *NF*-*κB*, *IL*-*6*, and *TNF*-*α* mRNA levels were significantly increased in hepatocytes in Groups F, L, and L + F compared with those in Group C. Moreover, 1,25(OH)_2_D_3_ reduced the high fat- or LPS-induced increase in *TLR4*, *NF*-*κB*, *IL*-*6*, and *TNF*-*α* mRNA levels in primary hepatocytes of Groups L, F, and L + F (*P* < 0.05; Fig. 4a–d). Western blot results showed that the levels of TLR4, phospho-NF-κB 65, and TNF-α were increased in Groups L, F, and L + F compared with those in Group C (*P* < 0.05). Additionally, the levels of the above transcripts were markedly suppressed by 1,25(OH)_2_D_3_ in primary hepatocytes (Fig. 4e).

## Discussion

Diabetes mellitus often co-exists with different metabolic-related syndromes, such as dyslipidemia, hypertension, and NAFLD. In our study, diabetic rats showed abnormal lipid profiles and increased serum ALT and CRP levels. Pathological results showed that obvious inflammation and fibrosis occurred in diabetic rats compared with normal rats. Moreover, results of immunohistochemical staining, RT-PCR, and western blotting showed consistent upregulation of TLR4, NF-κB, and TNF-α expression in the livers of diabetic rats.

Vitamin D deficiency is closely associated with many hepatic diseases, including NAFLD, alcoholic liver disease, viral hepatitis, and hepatocellular carcinoma, as well as liver transplantation [8–12]. Patients with biopsy-proven NAFLD had lower 25(OH)D_3_ concentrations than the matched controls, and 25(OH)D_3_ concentrations were lower in individuals with NASH than in those with simple steatosis [13], suggesting the need for evaluation of the possible protective role of vitamin D supplementation in the development and progression of NAFLD. Recently, Nakano et al. [14] found that sunlight therapy ameliorates hepatocyte inflammation and fibrosis by regulating lipid transfer/metabolic proteins and vitamin D_3_ status. Additionally, Roth et al. [8] showed that the severity of NAFLD in rats consuming a vitamin D-deficient diet is increased compared with that in rats receiving vitamin D supplementation. In Asia, a cross-sectional study of 6567 Korean men found that participants with higher levels of serum 25(OH)D_3_ showed a significantly reduced risk for NAFLD compared with the low 25(OH)D3 groups [15]. Sharifi et al. [16] divided 53 patients into two groups in a parallel, double-blind, placebo-controlled study. The patients were randomly allocated to receive either one oral pearl consisting of vitamin D3 (50,000 IU) or a placebo every 14 days for 4 months. This study showed that the median serum 25(OH)D_3_ concentration significantly increased in patients who received vitamin D. Additionally, improved vitamin D status led to amelioration of serum high-sensitivity CRP (hs-CRP) and malondialdehyde (MDA) abnormalities. These findings suggested that supplementation with vitamin D may help prevent hepatic diseases. Consistent with these findings, our results showed that high-dose 1,25(OH)_2_D_3_ administration could significantly protect against liver inflammation and fibrosis in diabetic rats. However, some larger, randomized, placebo-controlled trials are required to detect the effects of vitamin D supplementation for the treatment of NAFLD and to determine the optimal levels of vitamin D. Furthermore, we found that the expression of TLR4, NF-κB, and TNF-α was downregulated by 1,25(OH)_2_D_3_ in a dose-dependent manner. We also observed differences in CD68 staining among groups, indicating changes in Kupffer cell/macrophage numbers. Many of the changes observed in the liver were secondary to the changes in Kupffer cell numbers, and these alterations may mediate the anti-inflammatory effects of high-dose 1,25(OH)_2_D_3_ in the liver of diabetic rats. Because high-dose 1,25(OH)_2_D_3_ may lead to problems such as hypercalcemia, we also tested serum calcium and phosphate levels. Our results showed that the levels of serum calcium and phosphate in rats treated with 1,25(OH)_2_D_3_ were not significantly different compared with those in normal rats.

We also observed reduced LDL cholesterol levels in rats in the high-dose 1,25(OH)_2_D_3_ group. Recently, Eflekhari et al. [17] found that treatment with 0.5 µg of calcitriol (1,25(OH)_2_D_3_) per day in patients with T2DM resulted in significant reductions in total cholesterol, LDL cholesterol, TG, and serum MDA levels. Thus, high-dose 1,25(OH)_2_D_3_ may reduce oxidative stress and improve lipid profiles.

Hepatocytes account for the largest number of cells in the liver and are involved in biosynthetic and metabolic processes. These cells can take up and remove LPS from portal circulation. In many studies, activation of the TLR4-mediated inflammatory pathway in hepatocytes has been shown to play an important role in the early stages of NAFLD [18]. Therefore, in this study, we treated primary hepatocytes with high fat, LPS, and 1,25(OH)_2_D_3_, individually or in combination. Our results showed that high fat and LPS treatments significantly increased the expression of TLR4, NF-κB, and downstream inflammatory factors, while treatment with 1,25(OH)_2_D_3_ decreased the expression of TLR4, NF-κB, and inflammatory factors.

The present investigation demonstrated that 1,25(OH)_2_D_3_ administration could partially protect against liver inflammation and fibrosis in diabetic rats in vivo and in vitro. The protective effects of this molecule may be related to the downregulation of components of the TLR4-mediated inflammation pathway. Intake of vitamin D through diet or sun exposure is only one of the many variables determining the activity of this pathway. Other variables include D binding protein (DBP) levels, the local synthesis of 1α, 25(OH)_2_D_3_, and vitamin D receptor (VDR) expression. Severe liver disease decreases vitamin D hydroxylation and albumin and DBP production. All of these factors are linked to the low levels of 25(OH)D. In our experiments, we found that compared to that observed in the control group, levels of ALB in diabetic rats decreased significantly. As a limitation of this study, we do not have information on the serum concentrations of 25(OH)D_3_ and DBP in different groups of rats. Further studies will be required to assess the levels of 25(OH)D, VDR, and DBP in rats and to define the mechanisms involved in the downregulation of TLR4-mediated inflammation and the side effects of high-dose 1,25(OH)_2_D_3_ administration. These studies should provide valuable insights into the development of new tr eatments for diabetic liver injury.
